# Bibliometric study and visualization of cellular senescence associated with osteoarthritis from 2009 to 2023

**DOI:** 10.1097/MD.0000000000037611

**Published:** 2024-04-26

**Authors:** Xueting Ding, Jingrui Huang, Raorao Zhou, Xianda Che, Yiming Pang, Dan Liang, Chengyang Lu, Yuhao Zhuo, Fuyang Cao, Gaige Wu, Wenjin Li, Penghua Li, Litao Zhao, XueQin Rong, Pengcui Li, Chunfang Wang

**Affiliations:** a Department of Embryology, School of Basic Medical Sciences, Shanxi Medical University, Shanxi, China; b Animal Experiment Center, Shanxi Medical University, Shanxi, China; c Orthopaedics, The Second Hospital of Shanxi Medical University, Shanxi, China; d Key Laboratory of Bone and Soft Tissue Injury Repair, The Second Hospital of Shanxi Medical University, Shanxi, China; e Laboratory department, Fenyang Hospital of Shanxi Province, Shanxi, China; f Pain Department, The Third People's Hospital of Hainan Province, Hainan, China.

**Keywords:** bibliometrics, cellular senescence, chondrocytes, CiteSpace, osteoarthritis, Scimago Graphica, VOSviewer

## Abstract

**Background:**

Osteoarthritis is a common degenerative joint disease that is highly prevalent in the elderly population. Along with the occurrence of sports injuries, osteoarthritis is gradually showing a younger trend. Osteoarthritis has many causative factors, and its pathogenesis is currently unknown. Cellular senescence is a stable form of cell cycle arrest exhibited by cells in response to external stimuli and plays a role in a variety of diseases. And it is only in the last decade or so that cellular senescence has gradually become cross-linked with osteoarthritis. However, there is no comprehensive bibliometric analysis in this field. The aim of this study is to present the current status and research hotspots of cellular senescence in the field of osteoarthritis, and to predict the future trends of cellular senescence in osteoarthritis research from a bibliometric perspective.

**Methods:**

This study included 298 records of cellular senescence associated with osteoarthritis from 2009 to 2023, with data from the Web of Science Core Collection database. CiteSpace, Scimago Graphica software, VOSviewer, and the R package “bibliometrix” software were used to analyze regions, institutions, journals, authors, and keywords to predict recent trends in cellular senescence related to osteoarthritis research.

**Results:**

The number of publications related to cellular senescence associated with osteoarthritis is increasing year by year. China and the United States contribute more than 70% of the publications and are the mainstay of research in this field. Central South University is the most active institution with the largest number of publications. *International Journal of Molecular Sciences* is the most popular journal in the field with the largest number of publications, while *Osteoarthritis and Cartilage* is the most cited journal. Loeser, Richard F. is not only the most prolific author, but also the most frequently cited author, contributing greatly to the field.

**Conclusion:**

In the last decade or so, this is the first bibliometric study that systematically describes the current status and development trend of research on cellular senescence associated with osteoarthritis. The study comprehensively and systematically summarizes and concludes the research hotspots and development trends, providing valuable references for researchers in this field.

## 1. Introduction

Osteoarthritis (OA) is a common degenerative disease of the joints that occurs mostly in the elderly population. OA can lead to disability and impose a significant economic burden on individuals and society.^[1]^ The main pathologic features of OA are defects in articular cartilage, reduction of chondrocytes, and loss of cartilage matrix.^[2]^ The main clinical symptoms of OA include chronic pain, joint instability, stiffness, deformity, and imaging joint space narrowing.^[3]^ The pathogenesis of OA has not yet been studied. However, risk factors associated with the development of OA have been proposed, mainly including age, gender, obesity, metabolic diseases, osteoporosis, genetic factors, and joint injuries.^[4]^ Existing therapeutic strategies for OA also tend to focus on palliation; therefore, it is crucial to elucidate the pathogenesis of OA and pioneer new therapeutic strategies. Cellular senescence is a cellular fate, a stable form of cell cycle arrest exhibited in response to a variety of stimuli, which plays an important role in both pathophysiology and has been studied for many years.^[5]^ In the last decade or so, researchers have found that increased cellular senescence leads to OA.^[6]^ Senescent chondrocytes are also present in joint injuries, and chondrocyte senescence stimulates cartilage degeneration leading to OA.^[7]^ Aging induces metabolic abnormalities in cells, and over time, these metabolic abnormalities can lead to the development of OA. Senescent fibroblast-like synoviocytes, which were found to increase significantly with the progression of OA in patients and mouse models, regulate the autophagy-the transcription factor GATA4 axis to promote cellular senescence and OA progression by influencing autophagy-associated 7 mRNA via methyltransferase-like 3.^[8]^ RNA-binding protein 1 has been found to be associated with the self-renewal capacity and senescence process of human mesenchymal stem cells (MSCs). RNA-binding protein 1 interacts with the 4’ untranslated region of the Toll-like receptor 4, thereby alleviating cellular senescence and OA.^[9]^ Senescent cells exhibit cell cycle arrest and senescence-associated secretory phenotype (SASP).^[10]^ A hallmark of SASP is the secretion of proinflammatory cytokines such as IL-6, IL-17, IL-1β, oncostatin M and tumor necrosis factor.^[11]^ These SASP factors induce OA, including inflammation, decreased cell proliferation, and extracellular matrix degradation. Inhibition of SASP proinflammatory factor expression inhibits cellular senescence. For example, intra-articular injection of IL-17-neutralizing antibodies reduces joint degeneration and decreases expression of the aging marker Cdkn1a.^[12]^ Human MSC-derived exosomes effectively delay aging while reducing the transcription of aging markers and SASP factors.^[13]^ Therefore, senescence plays an important role in both the pathogenesis and treatment of OA, and the study of cellular senescence is important for the diagnosis and prevention of OA. Despite the growing interest in the topic of OA-related cellular senescence, a comprehensive and meaningful analysis of the publication trends in this research area is still highly underdeveloped, and it is crucial to understand the research hotspots, future directions, and current status of cellular senescence in OA, which urgently needs to be summarized.

In recent years, bibliometrics has been used for quantitative and qualitative analysis in specific fields to reveal research hotspots and research trends more clearly. Although, bibliometrics related to cellular senescence have been published by researchers.^[5]^ However, the current status and trend of research on OA-related cellular senescence have not been revealed. CiteSpace, VOSviewer, and the R package “Bibliometrics” are bibliometric tools commonly used in the medical field. Therefore, we use bibliometrics to demonstrate the current state of the field and predict research trends. In this paper, we synthesize and visualize the literature related to cellular senescence in OA over the past 2 decades (2009–2023), so as to predict future research directions and provide researchers with new perspectives.

## 2. Methods

### 2.1. Data source and search strategy

**Figure 1.**
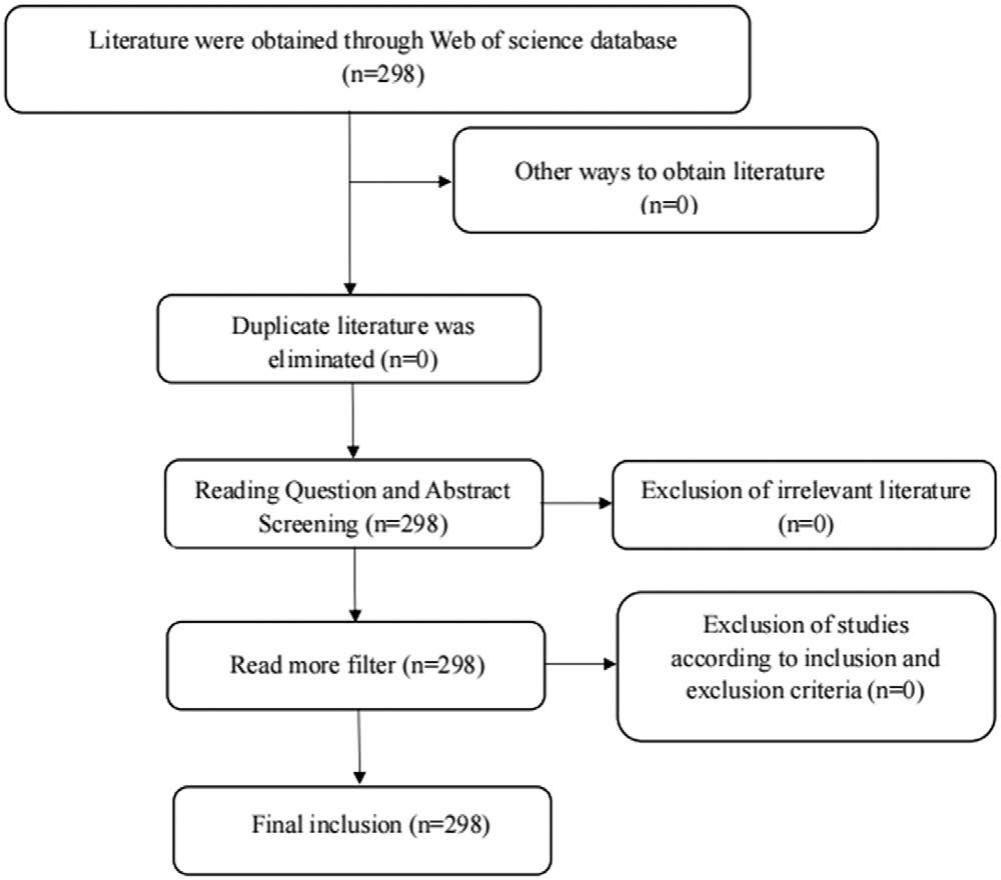
Flowchart of literature selection.

The Web of Science Core Collection database originating from Clarivate Analytics is considered to be one of the most authoritative and comprehensive database platforms hosting a wide range of international academic journals. Web of Science has been recognized and adopted by most previous research institutes as a high-quality repository of digital literature.^[14]^ Therefore, we chose it to obtain global academic information for bibliometric analysis. All published literature was extracted from web of science with a search date of January 1, 2009 to May 31, 2023. In this study, the search terms were as follows: Subject = Osteoarthritis* AND Topic = Cellular senescence AND Publication year = (2009–2023) AND Literature type = (article or review) AND Language = (English). In addition, all valid documentation, including year of publication, title, author’s name, nationality, and affiliation. As a result, 298 articles were retrieved (Fig. 1).

### 2.2. Analysis tool and parameter settings

We used Microsoft Office Excel software to graph trends in the number of articles and the percentage of national issuances and to summarize the data. VOSviewer and CiteSpace are considered to be the most commonly used tools for bibliometric analyses, VOSviewer maps authors, institutions, and national collaborative networks, etc, and CiteSpace 6.2 R4 software analyzes keywords and references. The R package “Bibliometrics” is also a commonly used bibliometric tool in the medical field, and was used in this study to complete the analysis of core journals and 3-field plots.^[15]^ In addition, the Scimago Graphica software analyzes country publications and collaborations to provide a more intuitive picture of how closely the country’s publication volume matches its collaborations.

## 3. Results

### 3.1. Search results

The search method is shown in Table 1. A total of 298 articles were retrieved using this search term, and 298 articles related to cellular senescence in OA were finally included in this analysis after duplicates were eliminated from the selected documents using the “Eliminate Duplicates” function in CiteSpace software.

**Table 1:** Summary of data source and selection.

Category	Specific standard requirements
Research database	Web of Science Core Collection
Searching period	January 2009–June 2023
Language	“English”
Searching keywords	TS=(“cell senescence” AND “OA”)
Document types	“Articles” AND “Review”
Data extraction	Export with full records and cited references in plain text format
Sample size	298

OA = osteoarthritis.

### 3.2. Overall performance of global literatures

**Figure 2.**
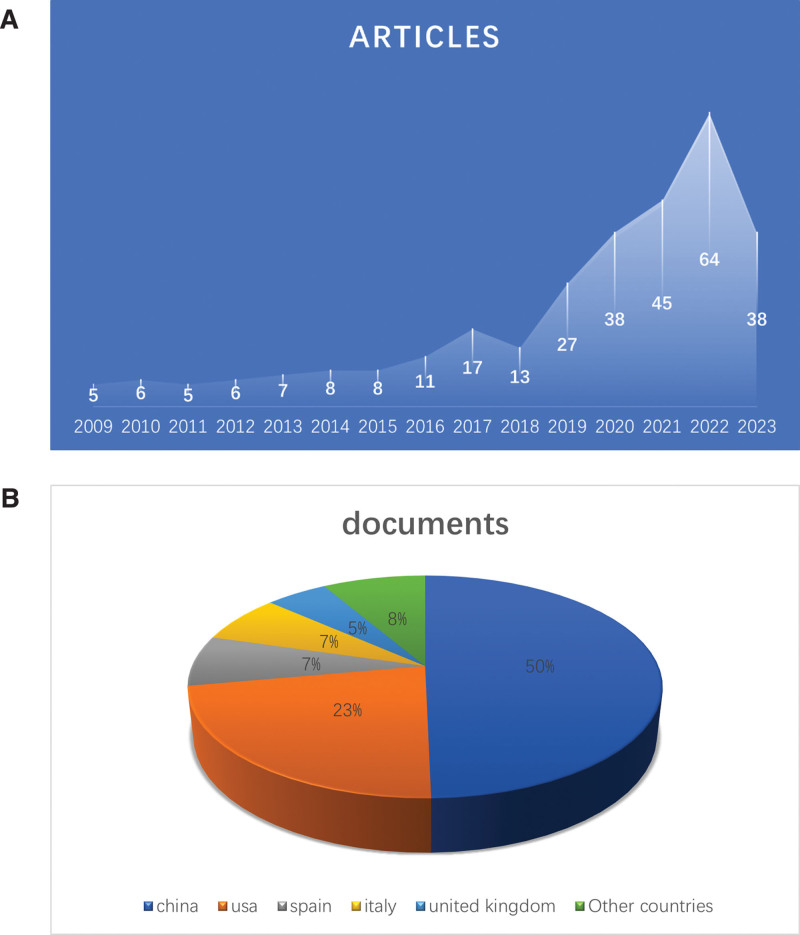
(A) Annual publication for cellular senescence in OA. (B) Proportion of national publications. OA = osteoarthritis.

The 298 papers included in this study came from 36 countries, 1802 authors from 466 institutions, published in 155 journals, and cited 13,024 papers from 2099 journals. The annual trend in the number of publications of OA-related cellular senescence studies (Fig. 2A) showed a steady annual increase in the global literature, surging from 13 in 2018 to 64 in 2022. China published the most papers (50%), followed by the United States (23%), Spain (7%), Italy (7%), and the United Kingdom (5%) (Fig. 2B). This indicates that the research on cellular senescence in the field of OA is accelerating, more and more scholars are focusing on the field, and cellular senescence has become a new focus of OA research.

### 3.3. Analysis of countries

**Figure 3.**
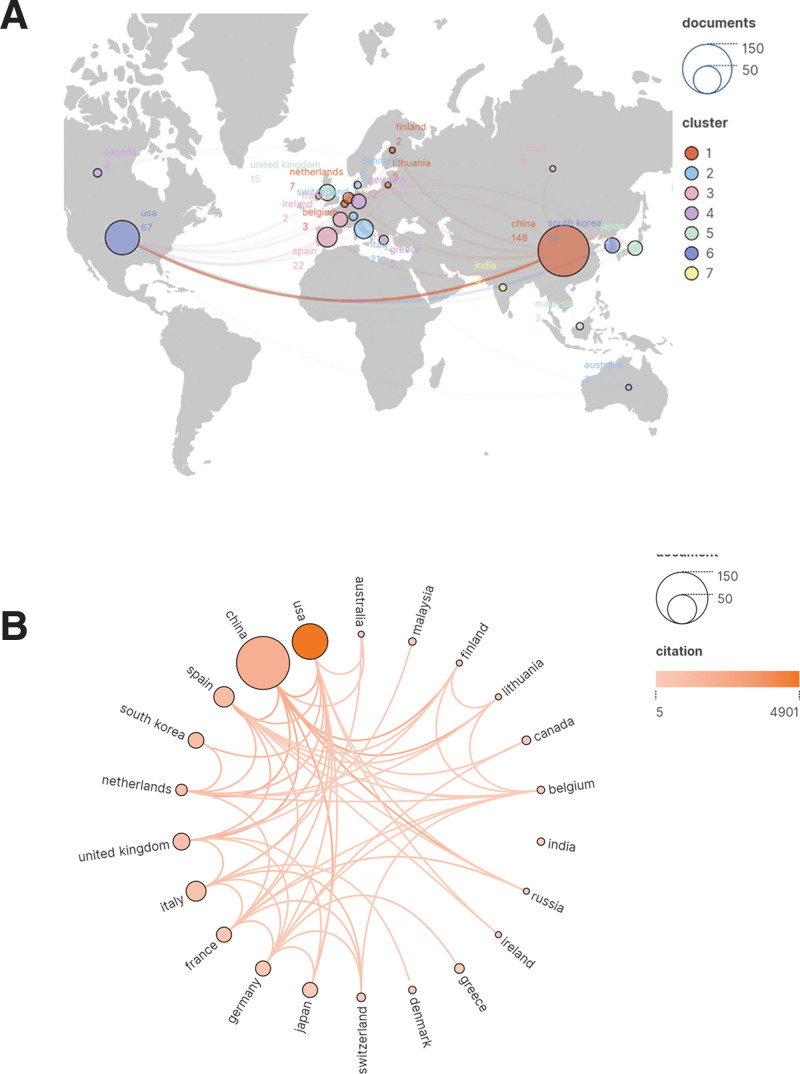
(A) Map of country publications and cooperation networks. (B) Citation visualization network atlases for cellular senescence studies in OA. OA = osteoarthritis.

Research in this area has been conducted in 36 countries, 22 of which have published more than 2 papers. A visual network mapping of country publications and citations constructed using Scimago Graphica software based on VOSviewr on the data (Fig. 3, A and B). Table 2 lists the top 5 countries in terms of publications and citations, with China (n = 148) being the most productive, followed by the United States (n = 67), Spain (n = 22), Italy (n = 21), and the United Kingdom (n = 15). Although the United States ranked second in terms of publications, it had the highest total citations, followed by China. In terms of citation frequency per citation, the United States had the highest average citation frequency for publications (73.1), followed by the United Kingdom (56.1), and Spain (52.3). The top 3 countries in terms of number of publications have formed close cooperation with other countries, which has contributed to the rapid development of research in this field.

**Table 2:** Top 5 countries in the education big data research field.

Rank	Country	Documents	Citations	Average citation/publication
1	China	148	1933	13.1
2	the United States	67	4901	73.1
3	Spain	22	1151	52.3
4	Italy	21	747	35.6
5	the United Kingdom	15	841	56.1

### 3.4. Analysis of institutions and authors

**Figure 4.**
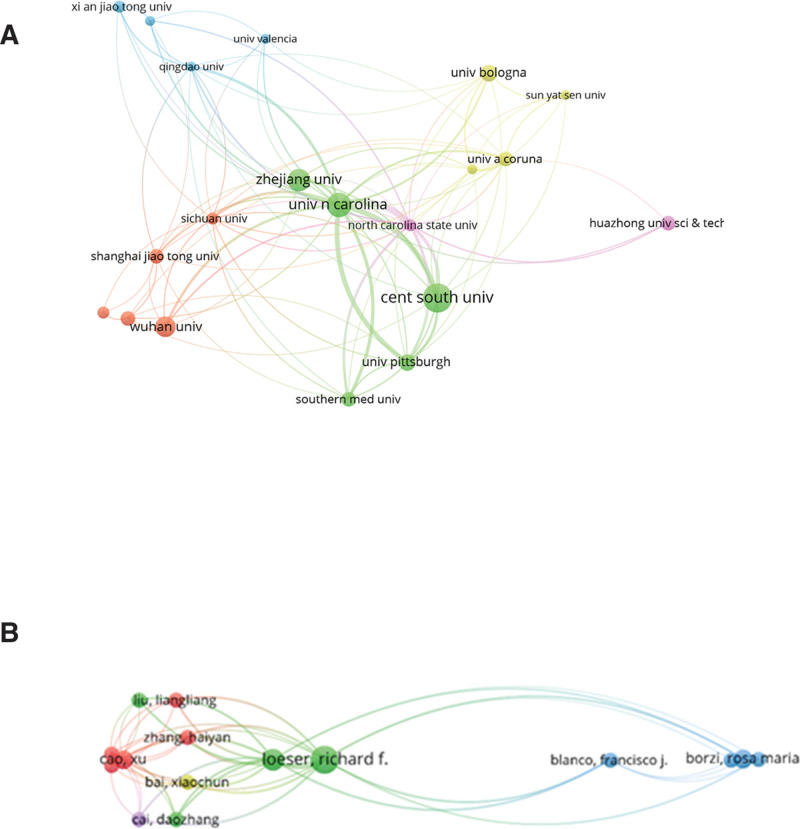
(A) Collaborative network of publishers of cellular aging research in OA. (B) Collaborative network of published authors of cellular senescence studies in OA. OA = osteoarthritis.

The Institutional Collaboration Network visualization in VOSviewer identifies the core institutions in the field, with the minimum publication threshold set at 5. A total of 20 institutions met the criteria. Figure 4A lists the top 20 contributing institutions and shows a network diagram of inter-institutional collaboration. The first-ranked institution is Central South University (n = 14), followed by University of North Carolina (n = 12) and Zhejiang University, which ranks third (n = 11). In addition, we found that there is a strong collaboration among several institutions such as Central South University, University of North Carolina, University of Pittsburgh, Zhejiang University, Wuhan University.

Table 3 lists the top 5 authors in terms of number of publications and citations. The top 5 authors contributed a total of 33 publications, or about 11% of all publications in the field. Loeser, Richard F., published the most research with 9 publications, followed by Diekman, Brian O., with 7 publications, Borzi, Rosa Maria, and Cao, Xu, with 6 publications each, and Blanco, Francisco J., with 5 publications (Table 3). Loeser, Richard F., as the highest-published author, also has the highest average citation rate with 6, and Blanco, Francisco J. with 5 (Table 3). Loeser, Richard F., as the author with the highest number of publications, also has the highest average citation rate, Diekman, Brian O. has the second highest average citation rate, and Blanco, Francisco J. is third. The author collaboration network visualization in VOSviewer identifies the core authors in the field and visualizes the collaboration between authors. Setting the minimum publication threshold to 5, a total of 15 authors met the criteria, and Figure 4B shows the collaborative network of 15 authors.

**Table 3:** Most important authors in the education big data research field.

Rank	Author	Documents	Citations	Average citation/publication
1	Richard F. Loeser	9	1663	184.8
2	Brian O. Diekman	7	880	125.7
3	Rosa Maria Borzi	6	308	51.3
4	Xu Cao	6	47	7.8
5	Francisco J. Blanco	5	588	117.6

### 3.5. Analysis of journals and research areas

**Figure 5.**
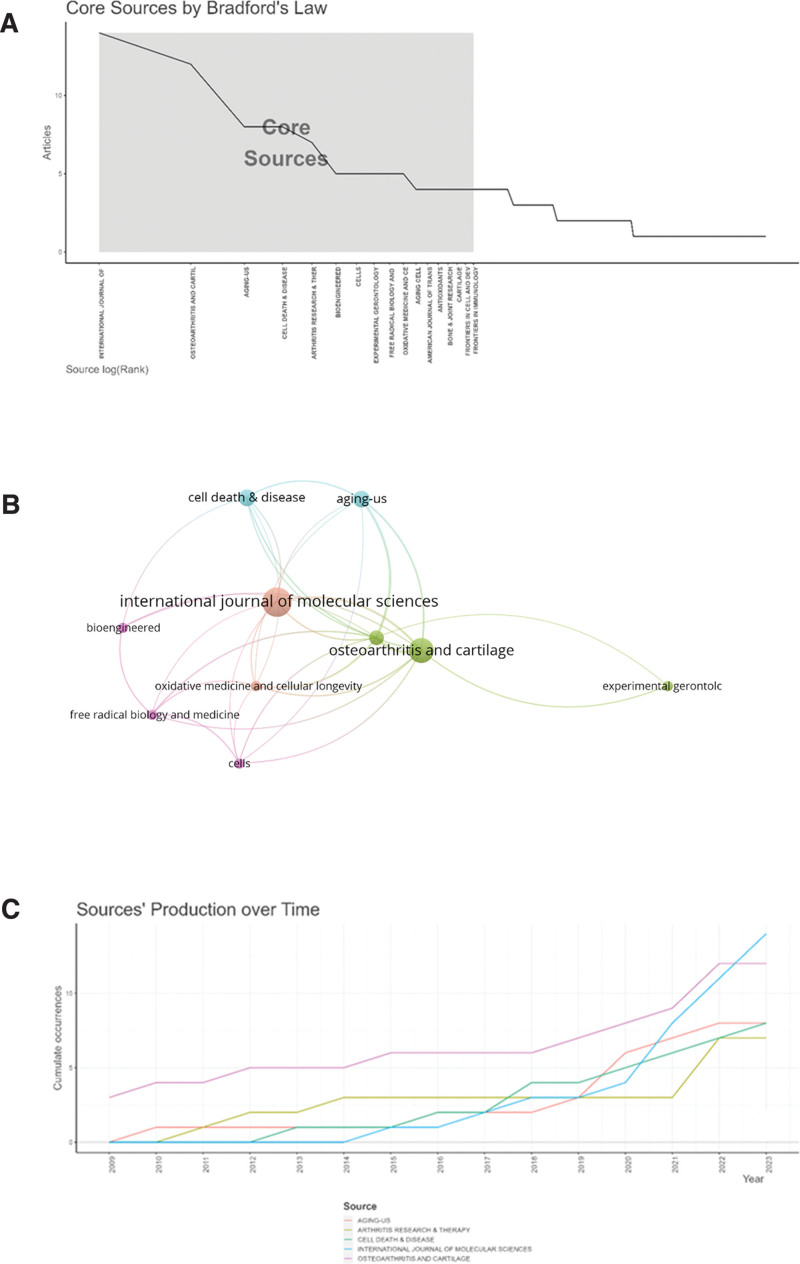
(A) Core sources by Bradford law. (B) Partnerships with journals in the top 10. (C) Source’ production over time.

Table 4 lists the top 10 productive journals involved in this study. *International Journal of Molecular Sciences, Osteoarthritis and Cartilage*, and *Aging-US* ranked in the top 3, with 14, 12, and 8 articles, respectively. The top 3 journals in terms of average citation rate are *Osteoarthritis and Cartilage, Free Radical Biology and Medicine, Oxidative Medicine and Cellular Longevity*. Bradford law is one of the most important laws of bibliometrics. Each scientific and technical journal belongs to a certain disciplinary classification, and if the scientific and technical journals are arranged in decreasing order according to the number of papers they publish specialized in their discipline, then the journals can be classified into core, related and unrelated zones dedicated to this discipline. In this paper, the core journals in the area were obtained through Bradford law (Fig. 5A). The names of the journals that were analyzed for co-citation using VOSviewer and defining the journals with a minimum of more than 5 citations are shown in Figure 5B. In addition, this paper also analyzed the scientific productivity of the top 5 journals in recent years (Fig. 5C), it is not difficult to find that the output of the top 5 journals has been rising, *Osteoarthritis and Cartilage* has always been high in the number of articles, *International Journal of Molecular Sciences* is the fastest growing, gradually overtaking *Osteoarthritis and Cartilage.*

**Table 4:** Top 10 journals in the education big data research field.

Rank	Source	Documents	Citations	Average citation/publication
1	*International Journal of Molecular Sciences*	14	366	26.1
2	*Osteoarthritis and Cartilage*	12	994	82.8
3	*Aging-US*	8	275	34.4
4	*Cell Death & Disease*	8	259	32.4
5	*Arthritis Research & Therapy*	7	212	30.3
6	*Bioengineered*	5	25	5.0
7	*Cells*	5	15	3.0
8	*Experimental Gerontology*	5	68	13.6
9	*Free Radical Biology and Medicine*	5	294	58.8
10	*Oxidative Medicine and Cellular Longevity*	5	186	37.2

### 3.6. Citation and co-citation analysis

**Figure 6.**
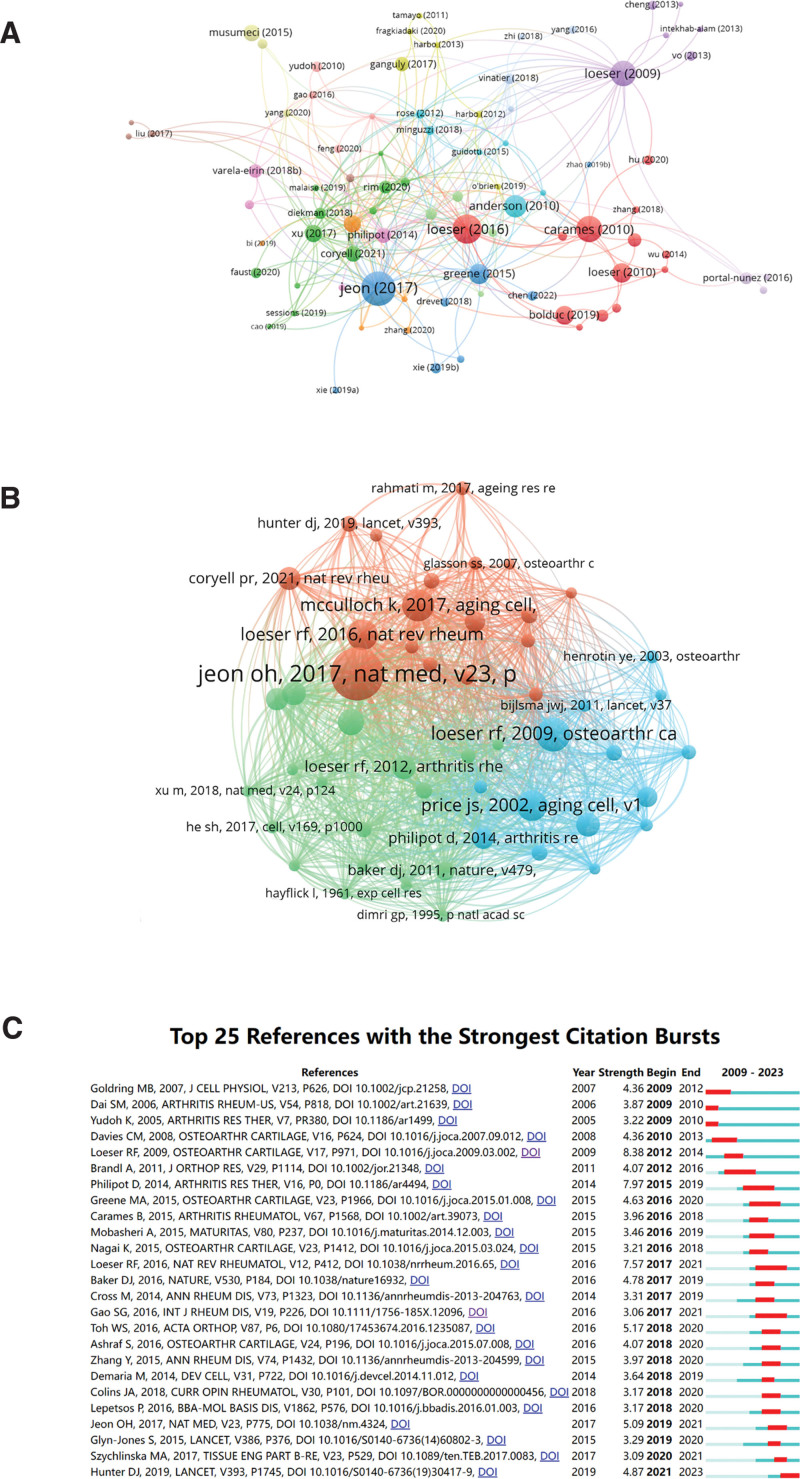
(A) Network map of citation analysis of documents with more than 20 citations. (B) Network map of co-citation analysis of references. (C) Top 25 most cited references in publications related to cellular senescence in osteoarthritis.

There are 97 articles in the field that have been cited more than 20 times (Fig. 6A). The top 10 most cited articles have been listed in Table 5. “Local clearance of senescent cells attenuates the development of posttraumatic OA and creates a pro-regenerative environment” was ranked first with 767 citations, second was “ Ageing and the pathogenesis of OA” with 570 citations, and third was “Autophagy is a protective mechanism in normal cartilage, and its aging-related loss is linked with cell death and osteoarthritis,” with citations of 461 times. In addition, it is not difficult to find out from the table that Loeser, Richard F. has published 6 of the top 10 cited articles as corresponding author, which is also consistent with Loeser, Richard F. being a top 1 scholar.

**Table 5:** The top 10 most cited articles in the field of cellular senescence and osteoarthritis.

Rank	Title	Corresponding author	Journal	IF	Publication year	Total citations
1	Local clearance of senescent cells attenuates the development of posttraumatic osteoarthritis and creates a pro-regenerative environment	Judith Campisi & Jennifer H. Elisseeff	*Nature Medicine*	82.9	2017	767
2	Ageing and the pathogenesis of osteoarthritis	Richard F. Loeser	*Nature Reviews Rheumatology*	33.7	2016	570
3	Autophagy is a protective mechanism in normal cartilage, and its aging-related loss is linked with cell death and osteoarthritis	Martin Lotz	*Arthritis & Rheumatology*	None	2010	461
4	Aging and osteoarthritis: the role of chondrocyte senescence and aging changes in the cartilage matrix	R. F. Loeser	*Osteoarthritis Cartilage*	7	2009	434
5	Why is osteoarthritis an age-related disease?	Richard F. Loeser	*Best Practice & Research Clinical Rheumatology*	5.2	2010	333
6	Aging-related inflammation in osteoarthritis	R. F. Loeser	*Osteoarthritis Cartilage*	7	2015	278
7	Age-related changes in the musculoskeletal system and the development of osteoarthritis	Richard F. Loeser	*Clinics in Geriatric Medicine*	3.3	2010	251
8	Reactive oxygen species, aging, and articular cartilage homeostasis	Richard F. Loeser	*Free Radical Biology and Medicine*	7.4	2019	229
9	Transplanted senescent cells induce an osteoarthritis-like condition in mice	James L. Kirkland	*The Journals of Gerontology®*	5.1	2017	197
10	Cellular senescence in osteoarthritis pathology	Taranjit Singh Rai	*Aging Cell*	7.8	2017	193

In addition, in Figure 6B, we analyzed co-cited references using VOSviewer to highlight the most influential ones. More importantly, citation burst is a valuable indicator of references of interest to scholars in the field over a period of time, and for this study, we used CiteSpace to identify the top 25 references with the strongest citation bursts (Fig. 6C), which cited citation bursts of citation durations. A 2009 publication entitled “ Aging and osteoarthritis: the role of chondrocyte senescence and aging changes in the cartilage matrix,” published in 2009, was ranked first (intensity = 8.38). In addition to this, articles published by Loeser, Richard F. and Gao, Shu-Guang were cited from 2017 through 2021.

### 3.7. Analysis of keywords and hotspots

**Figure 7.**
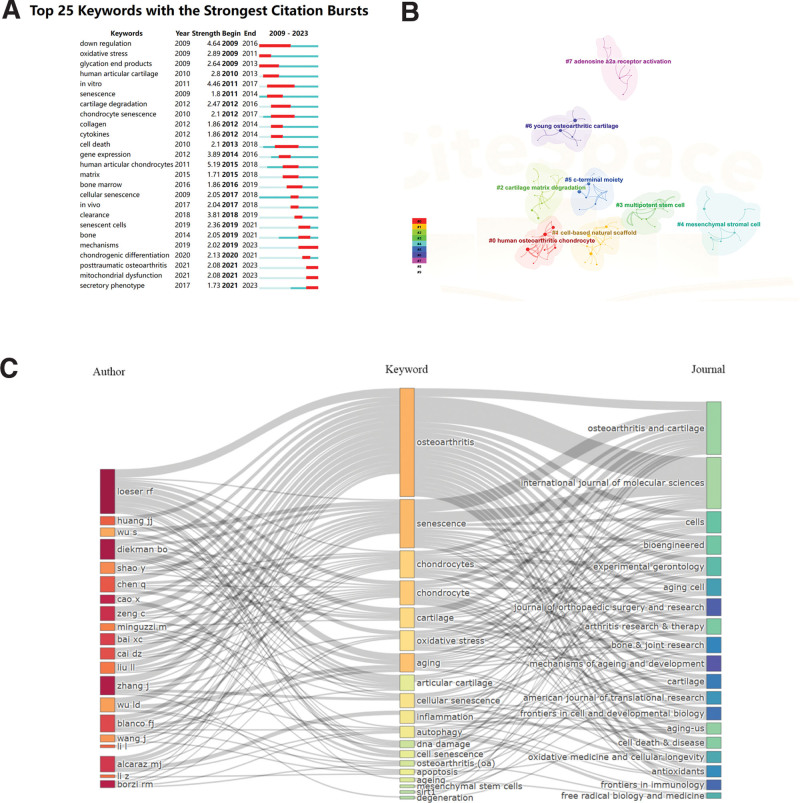
(A) Top 25 keywords with the strongest citation bursts based on CiteSpace. (B) Clustering analysis of the keyworks network based on CiteSpace. (C) Keywords plus 3-field graphical annotation for the analysis of cellular senescence in osteoarthritis: 3-field plot of the keywords analysis: (middle field: keywords; left field: authors; right field: journals).

CiteSpace’s algorithm was used to detect keyword bursts on top of burst detection. The top 19 keywords with the highest burst intensity (Fig. 7A). The keyword with the highest number of bursts was human articular chondrocytes (intensity = 5.19), followed by down regulation (4.64) and in vitro (4.46). The keyword with the longest outbreak was down regulation, which lasted for 8 years from 2009 to 2016. In recent years, chondrogenic differentiation, posttraumatic OA, secretory phenotype, and mitochondrial dysfunction have appeared in this field of research, suggesting that researchers are turning their attention to organelle The emergence of posttraumatic OA suggests that in addition to the focus on age as a key factor in the development of OA, posttraumatic OA is also becoming increasingly intertwined with cellular senescence. In addition to this, a network diagram was constructed in this study to visualize the keyword clustering (Fig. 7B), and it is easy to find that human osteoarthritic chondrocyte (cluster 0), cell-based natural scaffold (cluster 1), cartilage matrix degradation (cluster 2), multipotent stem cell (cluster 3), mesenchymal stromal cell (cluster 4), c-terminal motley (cluster 5), young osteoarthritic cartilage (cluster 6), and adenosine a2a receptor activation (cluster 7) are popular studies in the field since 2009, but the clusters are relatively fragmented from one another as research in the field has only begun in the last decade or so.

We created a 3-field diagram (Fig. 7C) showing the relationship between authors, keywords, and major elements of the journal by connecting the strengths of the links.^[16]^ OA, chondrocytes, and cartilage are consistent with Figure 6. The authors Loeser, Richard F. and Wu, S. established the relatively strongest link with the keywords “osteoarthritis” and “senescence.” In turn, the most significant links can be found with OA and senescence. In addition, it can be seen that OA and cartilage cover most of the links with the keywords “osteoarthritis,” “ senescence,” “chondrocytes,” “oxidative stress,” and “inflammation.” Therefore, it is clear from this visualization that cellular senescence is an important biological process that has significant implications for OA research.

**Figure 8.**
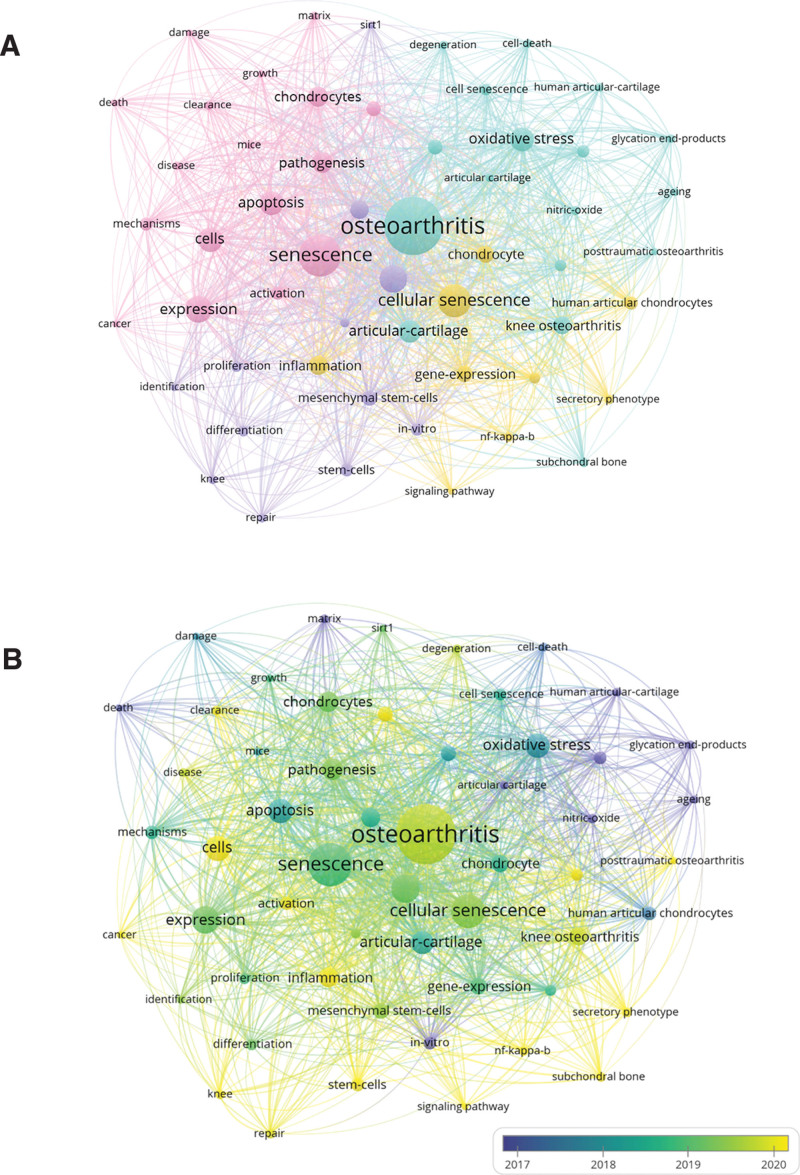
(A) Keyword mapping with cellular senescence in osteoarthritis; the frequency is expressed in dot size, and the keywords in the research field are divided into 4 categories: cluster 1: research on the mechanism of aging (pink), cluster 2: osteoarthritis research (green), cluster 3: restoration studies (purple), cluster 4: clinical research (yellow). (B) Distribution of keywords according to the mean frequency of appearance; keywords in yellow appeared later than those in blue.

In bibliometrics, keyword co-occurrence analysis is a common method to study popular research directions and areas, and has the function of monitoring and predicting the development of research in a particular field. For co-occurrence analysis, we used VOSviewer to perform co-occurrence analysis, and keywords were defined as words used more than 10 times in the titles or abstracts of all papers. In Figure 8A, the 55 keywords identified are mainly divided into the following 4 categories: cluster 1: study of aging mechanisms (pink), cluster 2: OA research (green), cluster 3: repair study (purple), cluster 4: clinical research (yellow). In 4 clusters, the research hotspots of cellular senescence in OA are presented. In the senescence mechanism research cluster, the main keywords used are aging, cellular, expression, pathogenesis. In the OA research cluster, the main keywords involved are OA, oxidative stress, articular cartilage, and knee OA. In the repair research cluster, commonly used keywords are cartilage, MSCs, aging, and proliferation. In the clinical research cluster, hot keywords are cellular senescence, inflammation, chondrocytes, and gene expression. The above 4 clusters are the most prominent research areas in OA-related cellular senescence.

In VOSviewer analysis, the frequency and time of occurrence of keywords determine the color of keywords. For example, keywords appear relatively early in blue and on the contrary in yellow. It is not difficult to find from Figure 8B that the research trend of most studies in the 4 clusters has transitioned from: aging mechanism study (cluster 1) and OA study (cluster 2) to repair study (cluster 3) and clinical study (cluster 4), indicating that researchers gradually discovered that cellular aging is an important pathological mechanism of OA. The gradual integration of cellular senescence and OA also shows that cellular senescence has become a new hotspot in the study of the pathological mechanism of OA and a new direction in clinical diagnosis and treatment.

## 4. Discussion

Researchers began focusing on cellular senescence at an early age and have been deeply involved in it for decades, with notable successes. It is only in the last decade or so that researchers have gradually linked OA to cellular senescence and have continued to explore it, giving new ideas for the study and diagnosis of OA.^[17]^ Autophagy,^[18]^ apoptosis,^[19]^ and mitochondrial dysfunction^[20]^ are all closely related to the development of OA, and the role that these biological processes play in the pathogenesis of OA has been meticulously explored by researchers. However, in the last decade or so, researchers have found that chondrocyte mitochondrial dysfunction, oxidative stress, and dysfunctional energy metabolism due to cellular senescence are inextricably linked to the development of OA.^[21]^ Therefore, the study related to cellular senescence in OA is a major research hotspot as well as a new research direction.

## 5. Basic information about the research

This paper uses bibliometric visualization to meticulously analyze and explore OA-associated cytosenescence for the first time. From Figure 2A, it is easy to find that the number of publications on OA-related cellular senescence has been increasing year by year and has surged since 2018. This phenomenon suggests that cellular senescence provides a novel perspective for exploring the pathogenesis and diagnostic strategies of OA.

Regarding countries, 36 countries have conducted research in this field, and each country has previously cooperated very closely. Throughout the world, several countries have conducted research in this field, among which, China has the largest number of publications, which indicates that China is the main force of research in this field (Fig. 3A). From Figure 3B, it can be seen that the top 3 countries in terms of the number of publications not only cooperate closely with each other, but also have close communication with other countries. However, in terms of the number of citations (Table 2), the average number of citations of the 3 countries, the United States, the United Kingdom, and Spain, ranked among the top 3, and the articles of these 3 countries have higher quality and persuasive power, which are recognized by most scholars.

In the analysis of institutions and authors, universities are still the main institutions that produce publications, and there is a deep collaboration between several universities (Fig. 4A). The institution with the highest number of publications is Central South University, indicating a greater emphasis on research in this area. In addition, we list the top 5 authors (Table 3). Loeser, Richard F. is not only the author with the highest number of publications, but also the author with the highest average number of citations, which suggests that Loeser, Richard F. is highly established in the field, with truly efficient and high-quality outputs, and with articles that are widely recognized. At the same time, Loeser, Richard F. has worked closely with many scholars (Fig. 4B), and is truly a top 1 scholar. It can be seen that communication and cooperation can promote research progress, and scientific research should carry forward the spirit of communication and cooperation.

This study lists the top 10 journals in terms of productivity according to Bradford law (Fig. 5A), where most of the journals are more oriented toward basic research. *International Journal of Molecular Sciences* is the number 1 journal in terms of number of articles, however, the journal with the highest average number of citations is *Osteoarthritis and Cartilage*, while *Osteoarthritis and Cartilage* also ranked second in terms of the number of articles, which indicates that the journal is not only highly productive but also widely recognized by researchers (Table 4). In addition, this paper also lists the publication volume of the top 5 journals in recent years, and it is not difficult to find that the publication volume of *Osteoarthritis and Cartilage* remains high, and the output of the *International Journal of Molecular Sciences* has been growing explosively (Fig. 5C).

## 6. Research hotspots and trends

This paper found through citation analysis (Fig. 6A) that the highest cited literature is related to the effect of cellular aging on the development and healing of posttraumatic OA, which has a high impact. It is pointed out that age is not a single cause of cellular aging, there will also be senescent cells in posttraumatic OA, the presence of senescent cells has a great impact on the development of posttraumatic OA, selective elimination of senescent cells will weaken the development of posttraumatic OA, reduce pain and increase cartilage development, under the dual influence of age and trauma, OA will be further aggravated.^[7]^ However, in the top 10 cited articles (Table 5), there are many reviews, and there are no research articles on mechanism and clinical efficacy, which may be related to the gradual association of cellular aging with OA in the past 10 years. The first 10 highly cited articles mainly include the relationship between cellular senescence and age,^[22]^ and the pathological role of cellular senescence in OA. The impact of aging and inflammation on OA is complementary, with age, the body will promote inflammation, the presence of inflammatory factors will aggravate cellular senescence, at the same time, one of the mechanisms of cellular aging to promote chronic inflammation,^[23]^ the relationship between cellular aging and inflammation and its impact on OA need to be further explored. Therefore, the study of the mechanism involved in cellular senescence in OA is a direction worthy of attention, which has become a hot topic in the field of OA, attracting a large number of researchers to study and need to be further explored.

The keyword co-occurrence showed that OA and aging were the largest nodes, representing the high popularity of aging in OA research. Articular cartilage, chondrocytes are the main research tissues in this field, and mice are commonly used laboratory animals in this field. There are multiple phenotypes of cellular senescence, 3 of which are senescence-associated β-galactosidase production, tumor suppressor protein the cyclin-dependent kinase inhibitor p16 (p16) expression, and extracellular vesicle secretion. Among them, joint cartilage aging is characterized by the presence of p16(INK4a)-expressing chondrocytes, tissue accumulation of p16(INK4a)-positive cells in the life cycle is harmful to tissue function, p16(INK4a) is sufficient to induce the production of 2 matrix remodeling enzymes MMP1 and MMP13, which shows that cellular senescence is closely related to OA pathogenesis.^[24]^ It is important to maintain the stability of joint cartilage and cartilage matrix, as senescent cells can lead to poor tissue ability to maintain homeostasis under stress, leading to the breakdown and loss of joint cartilage.^[25]^

The researchers used p16-3MR transgenic mice to selectively track and remove senescent cells after the ACL transection, and OA improved in the mice.^[7]^ After injecting senescent cells and nonsenescent cells into the knee joint of mice separately, mice injected with senescent cells showed the characteristics of OA, which, in addition to imaging features, were accompanied by increased pain sensitivity and changes in limb function.^[26]^ The removal of senescent cells and the injection of senescent cells in mice had very different reactions, which showed that the presence of senescent cells led to the development of OA. In the past 10 years, in the elaboration of the pathological mechanism of OA, cellular aging has gradually been linked with oxidative stress, proliferation, apoptosis, autophagy, differentiation, activation, cell death, and other biology, and these biological process abnormalities have promoted the occurrence and development of OA. Among them, autophagy is a very important biological process for maintaining chondrocytes homeostasis, and mitochondrial autophagy mediated by hypoxia-inducible factor-1α can alleviate OA.^[27]^ The loss of autophagy in senescent cells leads to cartilage deterioration, and stimulating autophagy can effectively delay cartilage deterioration.^[28]^ The same is true of mechanisms in fibroblast-like synovial cells.^[8]^

Over time, the emergence of keywords such as stem cells, subchondral bone, and repair indicates that damage repair of OA has attracted the attention of researchers. Based on keyword co-occurrence, we found that MSCs, stem cells have gradually emerged in recent years, MSCs have a certain repair effect on OA, however, researchers found that young MSCs have anti-inflammatory properties, while aging MSCs have proinflammatory properties.^[29]^ Aging of MSCs found in synovial tissue and subchondral bone marrow accelerates the development of OA.^[30]^ It is not difficult to find that the aging of MSCs has a significant impact on OA, and it is necessary to pay attention to the role of MSCs in OA. The study of MSCs and cellular senescence in associated cartilage repair,^[31,32]^ as well as the successful repair of cartilage and chondrocytes by miR-29b-5p delivery based on stem cell homing hydrogels,^[33]^ suggest that scholars are increasingly interested in aging-related cartilage damage repair and actively combine cellular senescence with tissue engineering. From the perspective of keyword outbreak intensity, posttraumatic OA, mitochondrial dysfunction, secretory phenotype, chondrogenic differentiation are emerging hot spots in this field in recent years, and it can be seen that cellular aging has a great impact on posttraumatic OA. In addition, the emergence of keywords such as mitochondrial dysfunction, secretory phenotype, and chondrogenic differentiation indicates that relevant research in this field is moving in a more microscopic direction.

## 7. Limitations

Despite the comprehensive analyses, the amount of data is still relatively small as cellular senescence has only been progressively cross-linked with OA for more than a decade, thus preventing a detailed analysis of the field. Since articles are updated daily, some newly published high-quality articles may have been overlooked. In addition, Web of Science Core Collection is the only database searched in this paper, which does not encompass all published articles, but has included the vast majority of the literature, and the database is internationally recognized as authoritative and credible. In conclusion, the analysis in this study can reveal the research hotspots and future trends of cellular senescence in OA.

## 8. Conclusion

In conclusion, this study provides a comprehensive picture of cellular senescence associated with OA and predicts future hotspots. Cellular senescence has been gradually associated with OA only after the 20th century, and researchers are gradually focusing on the impact of cellular senescence on OA, with the number of publications still increasing at a high rate. The communication and cooperation between the various teams and institutions should continue to be strengthened. In terms of the current state of research, cellular senescence is mostly associated with apoptosis, cell proliferation, autophagy, and other biological processes. The role of cellular senescence in posttraumatic OA has also attracted the interest of researchers, and the removal of senescent cells has a positive impact on OA. In terms of research trends, articular cartilage and chondrocytes are still the main focus of this field, and the removal of senescent cells is beneficial to the alleviation of OA. We also found that stem cells, subchondral bone, secretory phenotypes, cartilage repair, and mitochondrial dysfunction are becoming more and more popular in this field, and we will continue to explore this field in depth in order to find a more appropriate preventive and therapeutic strategy.

## Author contributions

**Data curation:** Xueting Ding, Jingrui Huang, Chengyang Lu.

**Software:** Xueting Ding, Yiming Pang, Yuhao Zhuo.

**Writing—original draft:** Xueting Ding.

**Writing—review & editing:** Xueting Ding, Pengcui Li, Chunfang Wang.

**Validation:** RaoRao Zhou, Wenjin Li.

**Methodology:** Xianda Che.

**Visualization:** Dan Liang.

**Investigation:** Fuyang Cao, Penghua Li, Litao Zhao.

**Conceptualization:** Gaige Wu, XueQin Rong.

**Funding acquisition:** Pengcui Li, Chunfang Wang.

## Supplementary Material


