# Supplementary material for: Bibliometric study and visualization of cellular senescence associated with osteoarthritis from 2009 to 2023
Source: Medicine (Baltimore). 2024 Apr 26;103(17):e37611. doi: 10.1097/MD.0000000000037611 (PMC11049721; doi:10.1097/MD.0000000000037611)
Supplement: Supplementary file 1 [file medi-103-e37611-s001.docx]

| **Abbreviations** | |
| --- | --- |
| Full name | Abridgement |
| Osteoarthritis | OA |
| the transcription factor GATA4 | GATA4 |
| autophagy-associated 7 | ATG7 |
| methyltransferase-like 3 | METTL3 |
| RNA-binding protein1 | PUM1 |
| mesenchymal stem cell | MSC |
| Toll-like receptor 4 | TLR4 |
| senescence-associated secretory phenotype | SASP |
| extracellular matrix | ECM |
| senescence-associated β-galactosidase | SA-β-gal |
| extracellular vesicle | EV |
| the Cyclin-Dependent Kinase Inhibitor p16 | P16 |
| hypoxia-inducible factor-1α | HIF-1α |
| fibroblast-like synovial cells | FLSs |
